# An Infrared Nanospectroscopy Technique for the Study of Electric-Field-Induced Molecular Dynamics

**DOI:** 10.1021/acs.nanolett.4c01387

**Published:** 2024-08-01

**Authors:** Maria Eleonora Temperini, Raffaella Polito, Tommaso Venanzi, Leonetta Baldassarre, Huatian Hu, Cristian Ciracì, Marialilia Pea, Andrea Notargiacomo, Francesco Mattioli, Michele Ortolani, Valeria Giliberti

**Affiliations:** † Department of Physics, Sapienza University of Rome, Piazzale Aldo Moro 5, I-00185 Roma, Italy; ‡ Center for Life Nano- & Neuro-Science, Istituto Italiano di Tecnologia, Viale Regina Elena 291, I-00161 Roma, Italy; ¶ Center for Biomolecular Nanotechnologies, Istituto Italiano di Tecnologia, Via Barsanti 14, I-73010 Arnesano, Italy; § Istituto di Fotonica e Nanotecnologie, Consiglio Nazionale delle Ricerche, Via del Fosso del Cavaliere 100, I-00133 Roma, Italy

**Keywords:** IR nanospectroscopy, electric-field-induced molecular dynamics, electrostatic AFM probe, vibrational Stark effect, membrane proteins

## Abstract

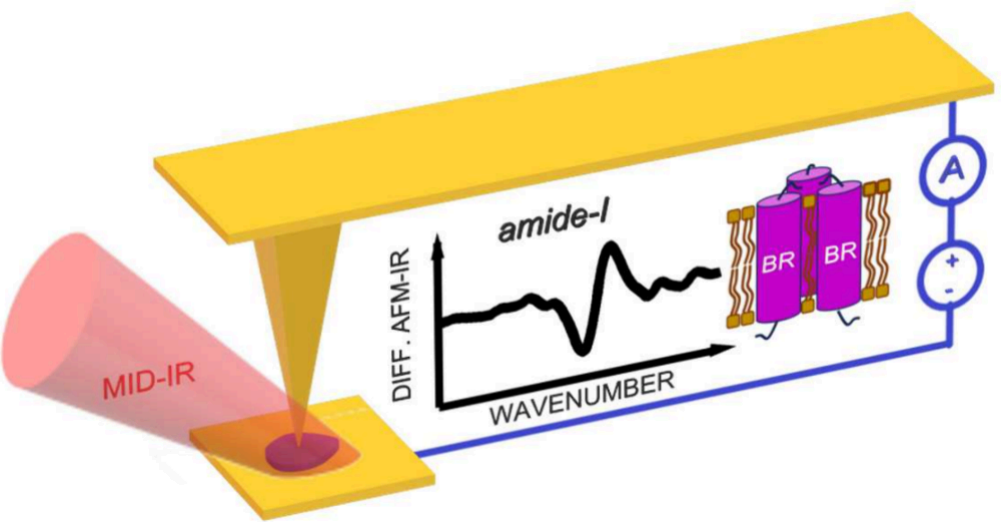

Static electric fields play a considerable role in a variety of molecular nanosystems as diverse as single-molecule junctions, molecules supporting electrostatic catalysis, and biological cell membranes incorporating proteins. External electric fields can be applied to nanoscale samples with a conductive atomic force microscopy (AFM) probe in contact mode, but typically, no structural information is retrieved. Here we combine photothermal expansion infrared (IR) nanospectroscopy with electrostatic AFM probes to measure nanometric volumes where the IR field enhancement and the static electric field overlap spatially. We leverage the vibrational Stark effect in the polymer poly(methyl methacrylate) for calibrating the local electric field strength. In the relevant case of membrane protein bacteriorhodopsin, we observe electric-field-induced changes of the protein backbone conformation and residue protonation state. The proposed technique also has the potential to measure DC currents and IR spectra simultaneously, insofar enabling the monitoring of the possible interplay between charge transport and other effects.

Static electric fields play a considerable role in a variety of molecular nanosystems as diverse as single-molecule junctions,^1−3^ molecules supporting electrostatic catalysis,^4−6^ and biological cell membranes incorporating membrane proteins.^7−9^ In these contexts, the use of nanoelectrodes, often in the form of conducting scanning probe tips, has been widely exploited to apply a controlled electric field to single molecules and ultrathin molecular layers.^3,10−16^ Depending on the specific molecular system, the application of an oriented static field results in diverse effects, such as charge transport,^1−3,17,18^ catalysis of chemical reactions,^4−6^ reorientation of intramolecular dipoles, modifications of the molecule conformation,^7,19−21^ and the shift of the vibrational energy levels known as the vibrational Stark effect (VSE).^22,23^ Although optical spectroscopies are, in principle, sensitive to most of these effects,^24−30^ rarely have they been performed in the near-field in the presence of a controlled external electric field. Using a conducting scanning probe both as nanoelectrode and as near-field optical sensor is then an ideal strategy to achieve this aim, since it allows one to apply a controlled electric field to a well-defined nanoscale volume. In few cases, Raman spectroscopy has been applied to nanosystems as a function of applied voltage bias, either in the form of tip-enhanced Raman spectroscopy (TERS) combined with scanning tunneling microscopy (STM)^31−35^ or by means of surface-enhanced Raman spectroscopy (SERS) configurations based on purposely designed metallic nanostructures.^36−41^ In the case of IR absorption spectroscopy, however, comparable nanoscale voltage-dependent vibrational studies have not been reported to date; however, there are classes of molecules for which IR spectroscopy becomes necessary, e.g., proteins. Indeed, the amide-I band between 1600 and 1700 cm^–1^ (photon energy of ∼0.2 eV) is a very sensitive IR spectroscopy fingerprint of subtle protein conformational changes and also of the protein backbone orientation, if compared to other optical spectroscopies.^42−45^ The interaction between proteins and an external field can lead to changes of their conformation, or it can affect their dynamical behavior with potential impact on their biological functions.^7,19−21^ Despite this, there are only a few experimental studies capable of resolving the conformational changes of proteins while retaining control over the actual electric field value and direction.^20,46,47^

In this work, we demonstrate an IR nanospectroscopy technique that achieves the simultaneous overlap of a static electric field and an enhanced IR probing field in the nanogap between a metallic scanning probe tip and a metal-coated substrate. The proposed approach relies on a simple but innovative concept: the combination of photothermal expansion IR nanospectroscopy (AFM-IR) in constant-contact AFM mode that reaches molecular monolayer sensitivity,^48^ with an electrostatic AFM probe that applies an external potential bias. To do this, we originally implemented in our setup an external conductive-AFM circuit,^49^ which however has never been combined with an IR nanospectroscopic approach. To validate the novel experimental configuration, we start by detecting the electric-field-dependent changes in the carbonyl stretching band of poly(methyl methacrylate) (PMMA) films, interpreting them in the theoretical framework of the VSE to calibrate the absolute electric field value. Then, we report relevant results on individual 10 nm-thick lipid bilayer membranes incorporating bacteriorhodopsin (BR), a prototype membrane protein regulating proton transport across the membrane^50^ and relevant also for nanoelectronics applications.^51^ We perform IR difference nanospectroscopy of the amide-I band to detect electric-field-induced conformational changes, which we compare spectroscopically to the better-known light-induced conformational changes of BR. Although not shown here due to the negligible DC current of the chosen molecular systems (PMMA and BR), a DC current/IR correlation study would be relevant for all those systems capable of electron transport,^49^ including proteins.^17,52−54^ One may notice that for all IR nanospectroscopy approaches based on noncontact AFM modes,^55,56^ a similar approach would be much more challenging to realize as the electric field intensity would be modulated by the tapping frequency and, in addition, one could not simultaneously measure the DC current.

Starting from an existing AFM-IR platform (*Bruker-Anasys nanoIR2*^24^) we have built an external electric circuit,^49^ as shown in Figure 1a (details of the electrical setup in the SI). We record AFM-IR spectra *A*(ν) at constant bias *V*_*i*_ and at zero bias and subsequently calculate *δA*(ν)_*i*_ = *A*(ν)|_*V*_*i*__ – *A*(ν)|_0_. The acquisition sequence is schematically reported in Figure 1b. For each *V*_*i*_ the acquisition is repeated ∼10 times and the IR difference absorption spectrum Δ*A*(ν)_*i*_ is obtained by average. The requirement of a conductive tip with IR field enhancement and suitable mechanical properties for AFM-IR translates into the choice of conducting silicon AFM probes with a pyramidal tip, coated with a gold layer added by evaporation. For our experimental purposes, we have further modified them to obtain a flat-top shape (width ∼80 nm) by melting the small portion of gold coating forming the tip apex, which we did by passing a high DC current through the probe while in contact mode with a conducting sample^49^ (see Figure 2a).

**Figure 1:**
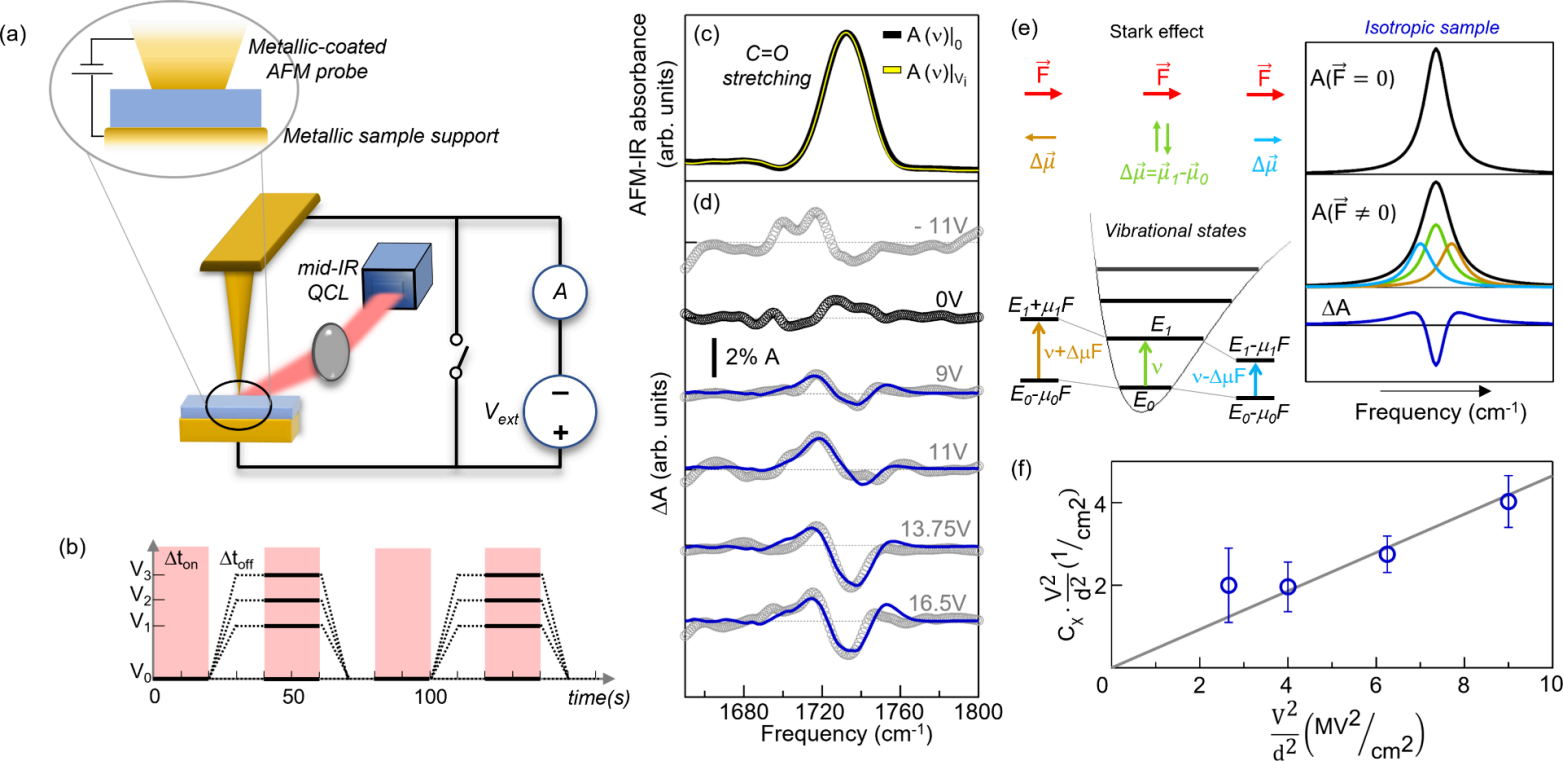
(a) Schematic of the DC circuit integrated in the AFM-IR setup. (b) Time scheme of the procedure followed for the acquisition of difference-spectroscopy data *δA*(ν)_*i*_. Δ*t*_*on*_ represents the time interval for the AFM-IR spectra acquisition (in Figures 1c and 2h,i Δ*t*_*on*_ ∼ 20 s), and Δ*t*_*off*_ represents the voltage ramp time plus a settle time for static charge removal (Δ*t*_*off*_ ∼ Δ*t*_*on*_). (c) Representative AFM-IR spectra recorded on the 55 nm-thick PMMA film with the flat tip in the absence (black) and in the presence (yellow) of an applied external voltage *V*. (d) Δ*A*(ν)_*i*_ acquired on the 55 nm-thick PMMA film for different values of the applied voltage *V*_*i*_ (gray dotted curves) and the best VSE fitting curves reported (blue lines). A smoothing spline algorithm has been applied to the AFM-IR data. (e) Schematic representation of the effect of an electric field on the vibrational transition energy in the case of an anharmonic molecular potential (left panel) and of the line shape broadening of an absorption peak for an isotropic sample because of the VSE (right panel). (f) Plot of the second derivative coefficients  (empty circles) obtained from the fitting curves in (d) using eq 1, highlighting the linear dependence on .

**Figure 2:**
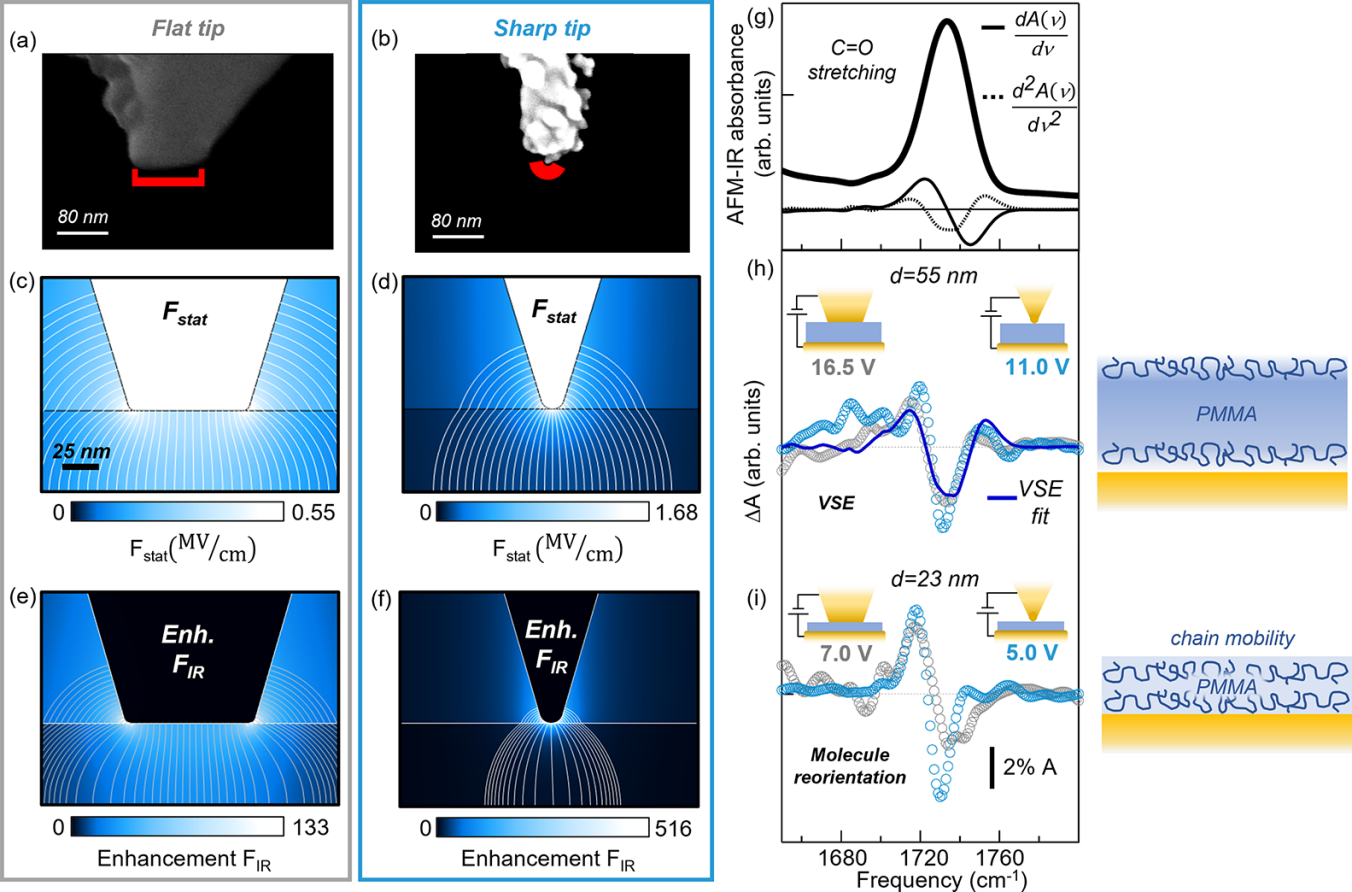
(a, b) SEM images of the flat tip and the sharp tip, respectively. (c, d) Numerical simulations of the static electric field *F*_*stat*_ applied with the flat tip and the sharp tip, respectively, on the 55 nm-thick PMMA sample for a voltage *V* = 1.0 V. (e, f) Electromagnetic simulations of the IR field *F*_*IR*_ enhancement at λ = 5.8 μm in the same experimental conditions of (c) and (d). The maps are saturated to the maximum field intensity in the PMMA layer. (g) Representative AFM-IR spectrum acquired on the 23 nm-thick sample with the flat tip and the relative first derivative (continuous black line, 30×) and second derivative (dashed black line, ×150). (h) Comparison of the difference spectra Δ*A*(ν)_*i*_ obtained with the flat tip (gray dotted curves) and (i) with the sharp tip (light-blue dotted curves) on the 55 nm-thick PMMA sample (top panel) and on the 23 nm-thick PMMA sample (bottom panel). A smoothing spline algorithm has been applied to the AFM-IR data.

PMMA can be used as the calibration sample because under the application of an external static electric field it shows modifications of the IR spectra due to a single effect, namely, the VSE.^57^ The VSE is the vibrational counterpart of the fundamental Stark effect, typically expressed in quantum mechanics as the frequency shift of an optical transition under the effect of an electric field, and it becomes a dominant effect when molecules have a negligible freedom of movement. In VSE, due to the anharmonicity of the molecular potential, the dipole moment *μ⃗* is slightly larger in the vibrational excited state than in the ground state; hence the corresponding energy levels shift differently in an electric field *F⃗*. As sketched in Figure 1e, left panel, this effect leads to a red or blue shift of the vibrational transition frequency Δν = −*F⃗* ·Δ*μ⃗*, where Δ*μ⃗* is the dipole moment change. In the case of isotropic molecule dipole moment distribution with respect to the field, as for PMMA films, neither a red or blue Stark shift is expected, but rather a line broadening proportional to the square of the static electric field (see Figure 1e, right panel).^22,58−60^ Note that, in principle, also the change of the electronic polarizability can contribute to the VSE, but this effect is typically negligible compared to that due to the change of dipole moment.^61^

PMMA films of two different thicknesses *d* = 55 and 23 nm were spin-coated over gold surfaces on conductive silicon wafers (see details in SI). In Figure 1c a typical AFM-IR spectrum of the C=O stretching line of PMMA is shown together with the Δ*A*(ν)_*i*_ curves for increasing *V*_*i*_ values in Figure 1d. Because *d* = 55 nm is smaller than the flat tip width of 80 nm, one can use the parallel-plate capacitor model to calculate the static applied electric field  and the VSE theory for an isotropic immobilized medium^22,62−64^ to fit the following equation to the data in Figure 1d (blue curves):
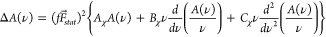
1where *f* is a local field correction factor between 1.0 and 2.0, χ is the angle between *F⃗*_*stat*_ and the radiation field *F⃗*_*IR*_, and *A*_χ_, *B*_χ_, and *C*_χ_ are molecular-bond-dependent coefficients. *B*_χ_ takes into account the field-dependent effect due to the change of polarizability, while *C*_χ_ is expected to be the VSE dominant contribution since *C*_χ_ ∝Δμ^2^. If we now plot the best-fit values of *C*_χ_·(*fF*_*stat*_)^2^ vs  (Figure 1f), we observe an approximate linear dependence because, in first-order perturbation theory, Δ*μ⃗* does not depend on the field strength. As detailed in SI, assuming *f* = 1.0, we find Δμ = (6.5 ± 0.6) × 10^–2^ Debye (electric dipole moment unit equal to 3.34 × 10^–30^ C·m), which is compatible with the values reported in refs (57) and (62). This is strong experimental evidence that a homogeneous field in the probed nanovolume with strength  is a good approximation for *d* = 55 nm, as also confirmed by the Poisson equation simulations of Figure 2c and the electromagnetic simulations of Figure 2e (details in SI). One can reasonably assume that the modification of the electric field value in the nanogap due to the photothermal expansion can be safely neglected.^65^

We have then explored the ultimate limits of field enhancement that can be reached with our technique, using AFM probes with a much sharper apex prepared by gold ion cluster evaporation (NextTip, Spain, SEM images in Figure 2b, red mark showing a curvature radius of 8 nm). Electrostatic and electromagnetic simulations of the sharp tip (Figure 2d,f) show an increase in both the static and IR field intensities at the tip apex, with a rapid decrease along the *z* axis. The nanoscale volume contributing to the difference signal is expected to be much smaller than that probed by the flat tip, with a radial extension set by the curvature radius of the tip, as is typical in near-field microscopy. In Figure 2h we compare the Δ*A* (ν)_*i*_ spectra measured on the 55 nm-thick calibration sample with the flat and sharp tips at *V*_*i*_ = 16.5 V and *V*_*i*_ = 11 V, respectively. One can see that the VSE line broadening observation is confirmed, but with *f* > 1 for the sharp tip, confirming the increase of the actual electric field in the probed volume. Interestingly, on a thinner PMMA sample (*d* = 23 nm, Figure 2i) the line shape of Δ*A*(ν) is qualitatively different compared to the *d* = 55 nm sample for both the flat and the sharp tip, resembling a first-derivative rather than a second-derivative signal (see Figure 2g). Indeed, for a PMMA film thickness comparable to the typical end-to-end distance of the polymer chain (around 22 nm in our case^66^), a large orientational freedom is expected^67,68^ and the results cannot anymore be interpreted in the framework of VSE. The observed Δ*A*(ν) featuring a first-derivative shape can therefore be ascribed to molecule reorientation that likely results in a change of the dipole–dipole interaction between the C=O bonds and consequent shift of the vibrational mode.^69,70^

We now move to the relevant case of proteins embedded in the lipid cell membrane, which represent the perfect prototype of molecules natively subjected to an electric field.^71^ It is known indeed that the transmembrane electric field exerts forces on the molecular structure, which can affect and even trigger functional conformational changes.^7,9,72^ We have selected the prototype proton-pump bacteriorhodopsin (BR) protein as a test sample to demonstrate a potential use of our technique.

We have prepared individual BR-containing purple membranes dispersed on ultraflat gold (details in SI), featuring a high BR-filling factor (75%). We recall that, in the BR molecule, seven antiparallel helix structures run from the extracellular (EC) to the cytoplasmic (CP) side of the cell membrane and that BR proteins are arranged in a hexagonal pattern of BR trimers. This results in a total C=O dipole moment *μ⃗* for the entire BR protein almost parallel to the normal to the membrane patch and a static dipole moment *μ⃗*_*stat*_ pointing from the CP side to the EC side (see simplified sketch in Figure 3a). In Figure 3 we report the results obtained on the BR sample with our novel setup on a selected stack of two purple membrane patches with a total thickness of 10 nm, as shown by the AFM topography map in Figure 3b. From surface roughness estimation by AFM imaging, one can conclude that the two stacked membrane patches are both oriented with the CP side up^75,76^ (see also SI). In Figure 3c we report a representative AFM-IR spectrum in the amide-I band range, which mainly arises from coupled C=O stretching along the protein backbone.^42^ The central frequency of this band (∼1665 cm^–1^) is in agreement with previous AFM-IR^24^ and s-SNOM^77^ papers, and it is a confirmation of the good quality of our samples in terms of protein arrangement within the lipid bilayer.^78^

**Figure 3:**
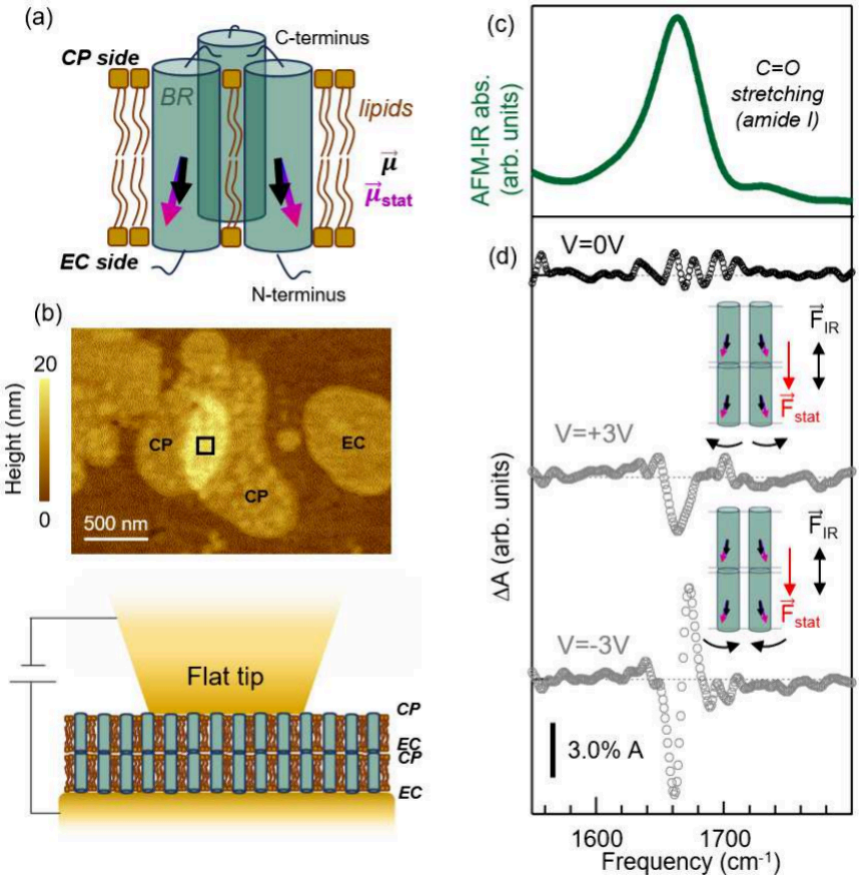
(a) Sketch reporting *μ⃗* and *μ⃗*_*stat*_ for two representative BR molecules belonging to the same trimer. The direction of *μ⃗*_*stat*_ has been obtained using the software described in ref (73) with PDB-ID Code 1FBB and that of *μ⃗* from ref (74). (b) Top: AFM topography of the two overlapping cell membranes exposing the CP side on which the AFM-IR measurement has been conducted. Cell membrane patches showing the less rough EC side can also be also identified. Bottom: sketch of two purple membranes placed in the nanogap between the flat tip and ultraflat gold substrate. (c) Amide-I band (green curve) of a representative AFM-IR spectrum acquired on the area pointed out by the black square in (b). (d) Δ*A*(ν)_0_ (black dotted curve) and Δ*A*(ν)_±3*V*_ (gray dotted curves) obtained in the double membrane of panel (b). A smoothing spline algorithm has been applied to the AFM-IR data.

Electric-field-induced IR absorption changes Δ*A* of a few percent of the maximum absorption peak at 1665 cm^–1^ (see Figure 3e) were obtained at bias *V*_*i*_ = ±3 V. These relative changes are comparable in intensity to those observed in the IR difference spectra of PMMA samples in Figures 1 and 2 at slightly higher *V*_*i*_. For the stack of membrane patches we can again assume, as for the PMMA films,  (no DC current was measured in the tip–substrate bias circuit, see SI). It is worth highlighting that, despite such high value of the electric field,^79,80^ no electroporation occurred during the experiments. This can be explained considering that the high protein-filling factor of BR is expected to result in an increase of the voltage breakdown for membranes.^80,81^ By looking at the Δ*A*(ν)_±3*V*_ reported in Figure 3d, one can clearly observe that very different features are obtained for the two polarities in the amide-I band range. Similarly to the case of 23 nm-thick PMMA film, here one can safely rule out the VSE as a dominant effect due to the large freedom of orientation of BR molecules^71^ (see also SI). To explain the observed amide-I features, we then have to resort to changes of the protein backbone orientation and/or conformation in external electric fields.^82,83^ Modifications of the individual helices (such as change of helix length, tilt, and partial unfolding), as well as of the intermolecular interactions, would all produce IR difference signals at the amide-I maximum.^42,78^ We believe that the dominant effect is the protein orientation, given the nonzero *μ⃗*_*stat*_ of BR proteins. BR orientation is expected to be different for opposite polarities due to two effects that break the dipole orientation symmetry under opposite *F⃗*_*stat*_ directions along the protein axis: the offset position of the center of *μ⃗*_*stat*_ toward the EC side with respect to the center of mass of the protein, and the different steric hindrance in the two orientation directions. Further contribution to the different Δ*A*(ν) is expected from the nonlinear dependence of the intermolecular coupling on the intermolecular and interhelix distances.^78^ In the SI we provide a phenomenological model pointing to different dominant effects, depending on the polarization of the applied bias. For *V*_*i*_ = +3 V, the negative dip is compatible with an increase of ∼1° between *μ⃗*_*stat*_ and *F⃗*_*IR*_ resulting in a reduction of the signal due to the selection rule, while the observed frequency shift of ∼9 cm^–1^ observed for *V*_*i*_ = −3 V is compatible with an increase of the intermolecular coupling due to the close-packing of the BR proteins and of the helices within each molecules.^78^ However, molecular dynamics calculations are required in order to identify the specific electric-field-induced changes of the BR backbone, but this is beyond the scope of the present work.

Finally, we have employed our technique to monitor the conformational changes of BR together with protonation changes of amino acids by keeping the membranes in high-hydration conditions to accumulate sufficient hydration water molecules in the proximity of the membrane stack.^84,85^ In Figure 4 we show the Δ*A*(ν)_−3*V*_ spectrum obtained in dark conditions on another individual purple membrane stack with the same CP-up orientation and high hydration. For comparison, in Figure 4 we also plot the light-induced IR difference spectrum previously acquired using the same AFM-IR setup without any static field applied but under visible light illumination.^24^ Both spectra display some protein conformational change features (yellow shade) and a peak at around ∼1760 cm^–1^ (red shade), which is the marker of the protonation change of individual amino acid residues. Obviously, the retinal photoisomerization and Schiff-base features (blue shade)^86^ appear only in the photocycle spectrum (green curve), which also explains the peak around ∼1760 cm^–1^, known to be related to the protonation of the amino acid Asp-85 acting as proton acceptor.^87^ The presence of the protonation change marker in the gray curve indicates a modulation of the protonation equilibrium of a protein residue, likely Asp-85, here induced by the electric force^88^ rather than by the absorption of visible photons. Interestingly, the protein conformational change features are quite different in the two cases. Also, the feature in the gray curve is different from the one observed in Figure 3d, with external electric field but in a condition of low hydration. Again, molecular dynamics simulations will help to clarify the nature of the protein backbone orientation in the presence of electric fields, with varying hydration conditions. In any case, our technique is perfectly suited to unambiguously measure electric-field-induced IR difference spectroscopy data on individual membranes, with known CP/EC orientation provided by simultaneous AFM imaging.

**Figure 4:**
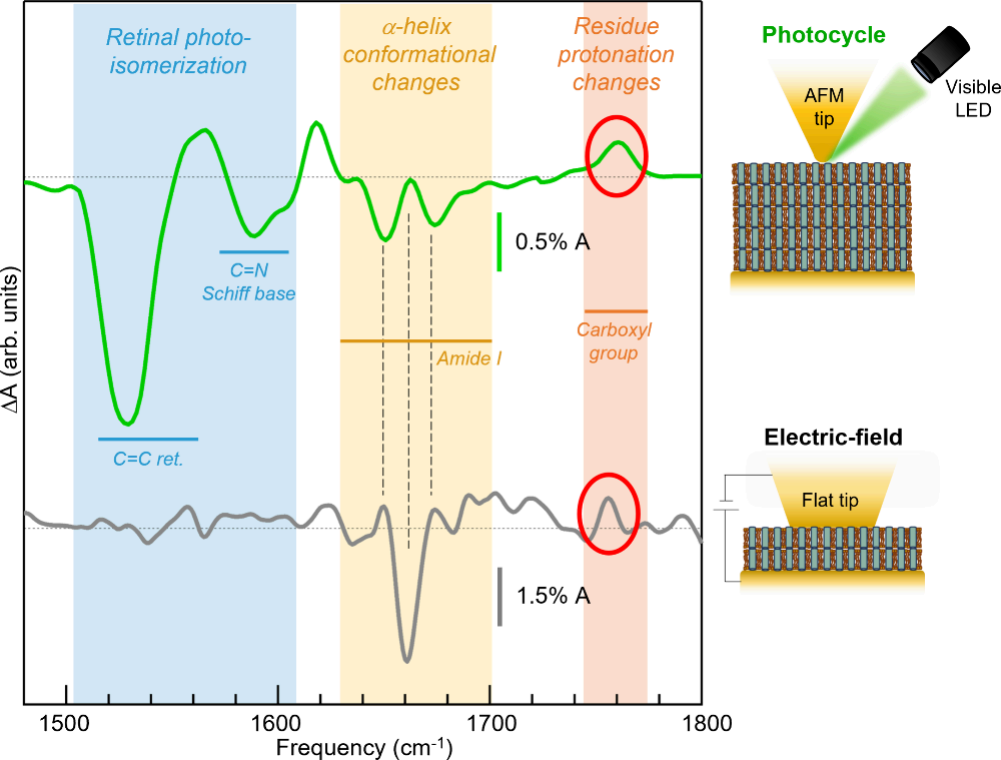
Comparison of two AFM-IR difference spectra (data smoothed with a spline algorithm). The green curve is the light-induced Δ*A* acquired on a purple membrane stack. The gray curve is an electric-field-induced Δ*A*(ν)_−3*V*_ acquired with a flat AFM tip on a stack of two overlapping purple membranes in high-hydration condition. The blue, yellow, and red shaded regions indicate the spectral range of the features related to retinal photoisomerization, protein conformational changes, and protonation of a carbonyl group, respectively.^86^

Beyond this preliminary experiment, future research along these lines may explain the dependence on an applied external electric field of the proton transport efficiency of BR-based photovoltaic devices,^89^ and, more in general, it may clarify the complex interplay between light-induced conformational changes, protonation dynamics of amino acid residues, and proton transport.^51,85^ One can envision that further improvements of the technique will allow to reach the sensitivity required to monitor spectral variations under electric field values closer to physiological ones ( rather than  achieved here for an individual membrane), to increase the biological relevance of the results.

In conclusion, we have demonstrated mid-infrared vibrational nanospectroscopy using metal-coated scanning probes in the presence of external electric fields. The probe tip apex in contact-AFM mode acts as both the nanoelectrode (with the metal-coated substrate acting as counterelectrode) and the IR field-enhancement structure, required to achieve nanoscale resolution in photothermal expansion spectroscopy. A sharper tip apex leads to higher electric field values, hence stronger IR difference signal for the same applied bias. We investigated the vibrational Stark effect in thin PMMA films and used the IR difference-spectroscopy data to calibrate the absolute electric field value, on the order of 3  for an applied voltage bias of 10 to 3 V depending on sample thickness, using a flat tip. On individual 10 nm-thick cell membrane stacks containing the proton-transport protein bacteriorhodopsin, we observed the field-induced conformational changes and the protonation of amino acid residues. The proposed technique permits one to apply well-defined and homogeneous electric fields to the controlled nanoscale volumes probed by infrared nanospectroscopy, and as such, it may find application in molecular electronics, electrostatic catalysis, fundamental studies of an electric potential on the cell transmembrane proteins, and protein studies in general.

## Supporting Information Available

Additional experimental details, materials, and methods including electromagnetic simulations (PDF)
