# Supplementary material for: An Infrared Nanospectroscopy Technique for the Study of Electric-Field-Induced Molecular Dynamics
Source: Nano Lett. 2024 Aug 1;24(32):9808–15. doi: 10.1021/acs.nanolett.4c01387 (PMC11328210; doi:10.1021/acs.nanolett.4c01387)
Supplement: Supplementary file 1 — nl4c01387_si_001.pdf [file nl4c01387_si_001.pdf]

Supporting Information for An Infrared Nanospectroscopy Technique for the Study of Electric-field Induced Molecular Dynamics

Maria Eleonora Temperini,[†,‡] Raffaella Polito,[†] Tommaso Venanzi,[‡] Leonetta Baldassarre,[†] Huatian Hu,[¶] Cristian Ciracì,[¶] Marialilia Pea,[§] Andrea Notargiacomo,[§] Francesco Mattioli,[§] Michele Ortolani,[†,‡] and Valeria Giliberti[*,‡]

†*Department of Physics, Sapienza University of Rome, Piazzale Aldo Moro 5, I-00185 Roma, Italy*

‡*Center for Life Nano- & Neuro-Science, Istituto Italiano di Tecnologia, Viale Regina Elena 291, I-00161 Roma, Italy*

¶*Center for Biomolecular Nanotechnologies, Istituto Italiano di Tecnologia, Via Barsanti 14, I-73010 Arnesano, Italy*

§*Istituto di Fotonica e Nanotecnologie, Consiglio Nazionale delle Ricerche, Via del Fosso del Cavaliere 100, I-00133 Roma, Italy*

E-mail: valeria.giliberti@iit.it

S1. Experimental setup

The AFM-IR platform (NanoIR2 by Bruker-Anasys) is based on the coupling of an AFM working in contact-mode and a broadly tunable quantum cascade laser (MIRcat, by Daylight

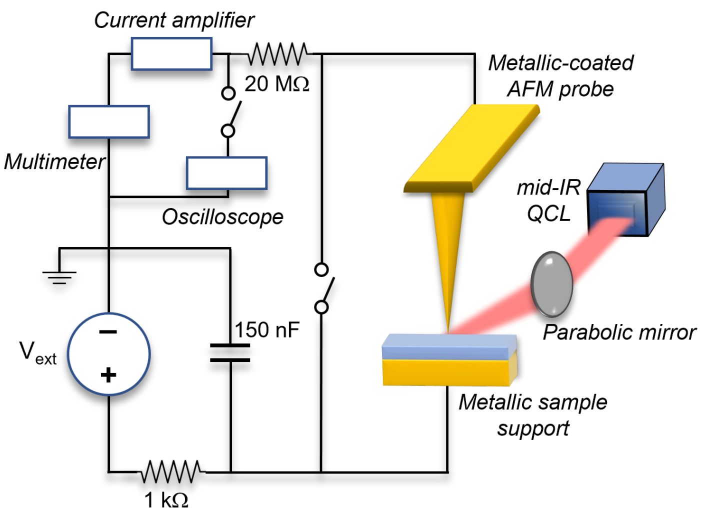

Figure S 1: Schematic of the DC circuit implementend in the AFM-IR setup.

Solutions) emitting in the 900-1900 cm^{-1} range. Such platform has been implemented with an external electric circuit to apply a DC bias exploiting metallic AFM probes, as shown in detail in Figure S1. The voltage source is a digital one (Keithley 2400 Standard Series SMU) and a current amplifier (DLPCA-200 by Femto) is inserted for current measurements. A by-pass closed during the approach of the tip to the sample together with a shunt resistance and capacitance avoid electric discharge trough the conductive tip. An oscilloscope allows to verify the formation of the electric contact between the tip and sample and it also serves to monitor accidental dielectric breakdown and Au melting events during experiments.

In this work we use two different AFM probes: 1) Contact mode NIR2 probes, model PR-EX-nIR2-10 by *Anasys-Bruker*. They are made of a silicon bulk cantilever and the tip has a pyramidal shape with gold layer added by evaporation. A SEM image is reported in Figure S2a. For our experimental purposes, the tip has been modified with a flat-top shape obtained by melting the small portion of gold coating forming the tip apex (Figure S2b) by passing a DC current through the probe while in contact-mode. SEM analysis has demonstrated that the gold coating likely extends over the entire flat top. 2) Contact mode sharp tip probes, model NT-IR-P-75 by *Next Tip*. They are silicon bulk pyramidal probes with a gold ion-cluster coating that permits to have curvature radius of the order of 10 nm

a the very tip apex (Figure 2b in the main text).

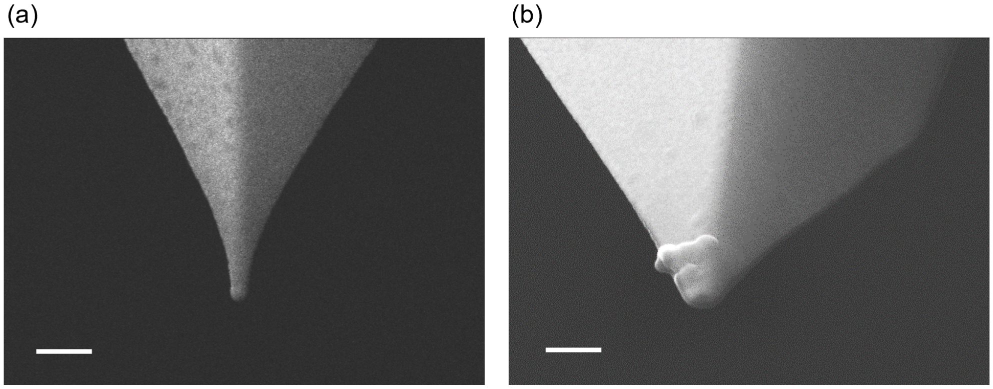

Figure S 2: (a) SEM images of a new NIR2 probe and (b) of a modified one after the melting process of the tip apex. Scale bar: 200 nm.

S2. Sample preparation

The sample substrates are made of highly doped (100) silicon (boron doped, resistivity: 0.001-0.005 $\frac{\Omega}{cm}$), covered with a thin adhesion layer of evaporated titanium (100 Å) followed by an evaporated gold layer (800 Å).

PMMA films A 6% PMMA (All Resist AR-P 669.06) was diluted in ethil-lactate to obtain a 1% solution which was spin-coated on the conductive substrate at different rotation speed resulting in different values of the film thickness d. Solvent evaporation was promoted using a hot plate heating step at T=170°C for 5 minutes. Two samples with different thickness of PMMA have been realized: d=23nm (3000 rpm) and d=55nm (obtained after 2 subsequent coating processes at 2000 rpm). The sample thickness was measured with AFM mapping in a purposely scratched area of the film.

Bacteriorhodopsin sample Purple membrane patches filled with Bacteriorhodopsin mutant D96N stored in buffer solution (20×10^{-3} M Bis-Tris propane, 100×10^{-3} M NaCl, 1×10^{-3} M $MgCl_2$, pH=6) were cast on the substrate and rinsed with milli-Q water after 5 minutes to remove the excess of salts and promote the adhesion of single and isolated membrane patches to the surface.

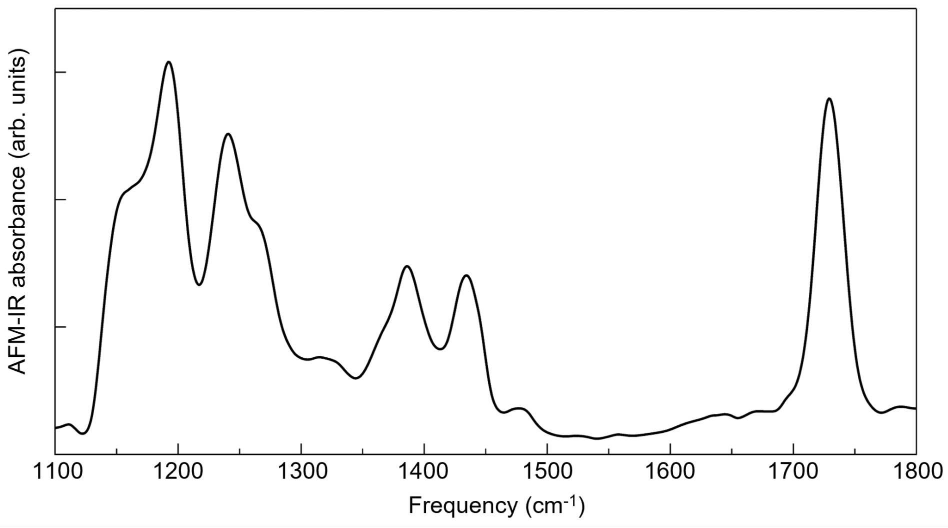

Figure S 3: AFM-IR spectrum of the 55nm-thick PMMA sample

S3. Additional AFM-IR spectra

In Figure S3 we report a representative AFM-IR spectrum of PMMA acquired in the 1100-1800 cm^{-1} frequency range.

S4. Simulations

In Figure S4 we report the numerical simulations for the static electric field F_{stat} and the enhancement of the IR field F_{IR} for the PMMA thicknesses d=55 nm and 23nm. We used COMSOL Multiphysics v6.1 to simulate the electrostatic and electromagnetic fields in the experiment. For the simulation of the electrostatic field, an axis-symmetric model was used since the tip was assumed to have a perfect cone shape with rotation symmetry. The AFM tips have an angle of 17 degrees. For the flat tip, the apex was truncated by a flat bottom facet with 80 nm diameters and for the sharp a tip radius of 8 nm was set as estimated from the SEM images. The molecular spacer was set to 23 nm and 55 nm with a permittivity of 2.1. Then applying voltage on the tip was set using boundary condition with 1 V electric potential, and the metal substrate on the bottom was set as the ground. With the electro-

static module solving for the stationary solution, we could obtain the electrostatic electric field with different spacer thicknesses. For simulating the IR field enhancement, similarly, we used the 2.5D simulation where the same axis-symmetric geometry is built but the electromagnetic wave equation was solved. Within the 2.5D simulation, the oblique incident plane wave (70 degrees to the surface normal) together with all vector fields could be decomposed in terms of the azimuthal mode number m by expanding the fields with cylindrical harmonics.[1] After summing up all the orders of modes that were simulated, we could reconstruct the total vector field from each cylindrical harmonic. A convergence check was implemented by increasing the number of the order m in the simulation. In the simulation, the incident wavelength was set to be 5.8 μm. The permittivity of gold was extracted from Olmon et al.[2] The IR field enhancement is obtained by comparing the IR field with the incident field, i.e., $\frac{|F_{IR}|}{|F_0|}$.

S5. Estimation of $\Delta\vec{\mu}_{C=O}$ in PMMA sample

Since $C_\chi \propto |\Delta\vec{\mu}|^2$, we can retrieve an estimation of $\Delta\vec{\mu}$ for the C=O bond from the values of C_χ obtained fitting the experimental data. In particular, $C_\chi = \frac{1}{30h^2c^2}|\Delta\vec{\mu}|^2[5 + (3cos^2\chi - 1)(3cos^2\zeta - 1)]$, where χ is the angle between the direction of the IR electric field F_{IR} and the static electric field F_{stat}, ζ is the angle between $\Delta\vec{\mu}$ and the transition dipole moment $\vec{M}$, h is the Planck constant and c the speed of light.[3] Considering $\chi = 0$ and $\zeta = 0$, one obtains $C_\chi = \frac{9|\Delta\vec{\mu}|^2}{30h^2c^2}$. Indeed, in our experimental condition we assume the angle χ equal to 0 because the IR electric field and the static electric field have the same direction in the probed volume under the tip, as also confirmed by the electromagnetic simulations reported in Figure S4 and we also assume $\zeta = 0$ as in Ref.[4] From the experimental data collected with the flat tip on the PMMA film with thickness d=55 nm, we obtained an average value of $C_\chi = 0.36 \pm 0.06\frac{1}{MV^2}$ and so $\Delta\vec{\mu} = 6.5 \pm 0.6 \cdot 10^{-2}D$.

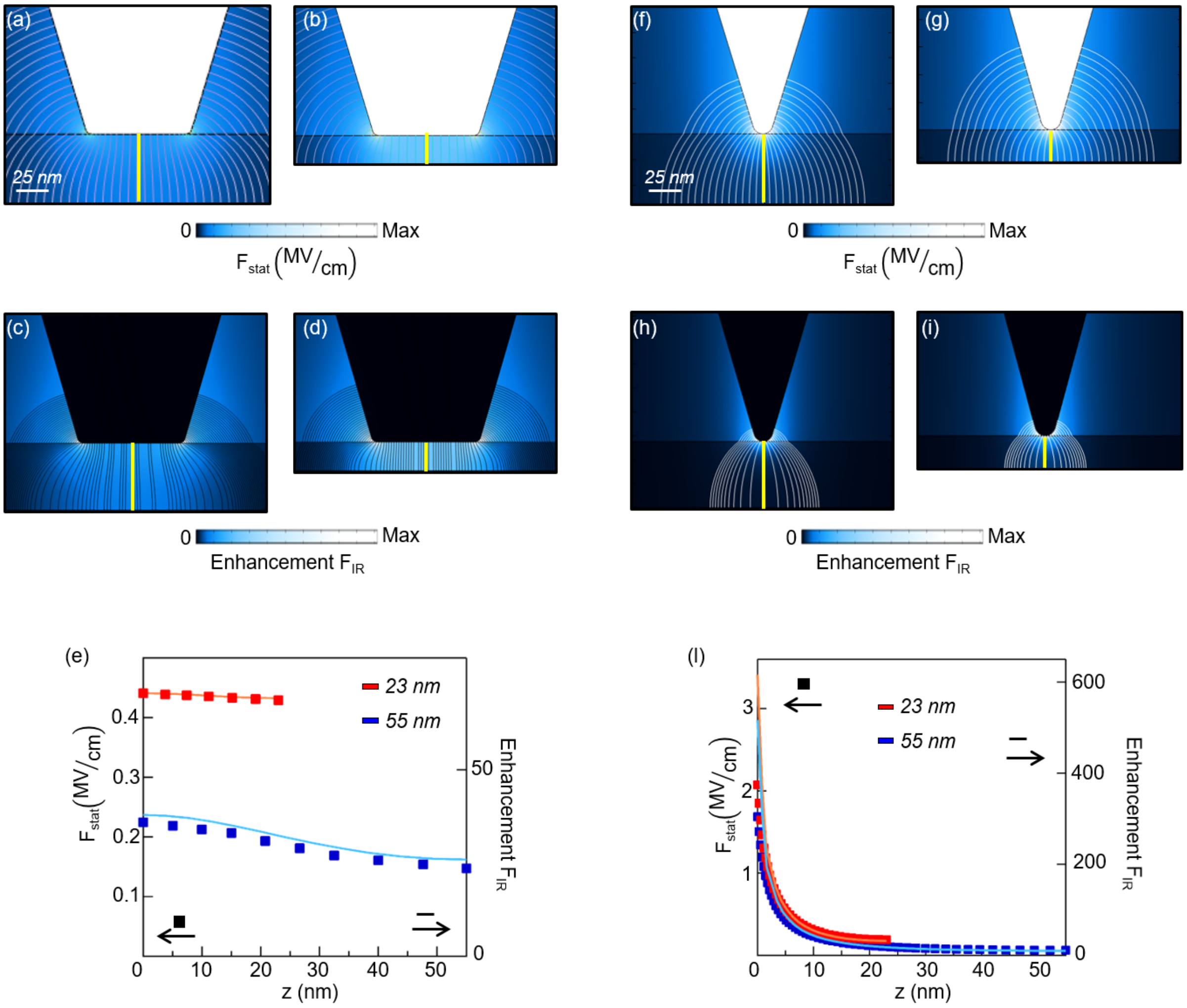

Figure S 4: Flat tip calculated maps: (a) and (b) static electric field simulations for an applied voltage of 1.0 V on the PMMA sample of thickness 55 nm and 23 nm, respectively. (c) and (d) Electromagnetic simulation of the IR field enhancement at λ= 5.8 μm for the PMMA sample of thickness 55 nm and 23 nm, respectively. (e) Profiles of the static electric field (squares, left axis scale) and of IR field enhancement (continuous lines, right axis scale) extracted from the simulations in (a), (b), (c) and (d) along the yellow line direction for the two thicknesses of the sample. Sharp tip calculated maps: (f) and (g) same of (a) and (b). (h) and (i) same of (c) and (d). (l) same of (e), extracted from the simulations with the sharp tip in (f), (g), (h) and (i).

S6. Topographic differences between CP and EC membrane side

In Figure S5 we show examples of AFM maps of purple membranes, better clarifying the surface topographic difference between the two sides of purple membrane (cytoplasmatic

(CP) or extracellular (EC) side), in agreement with literature.[5,6] Values of RMS roughness and mean roughness obtained for the EC (CP) side of purple membrane are: RMS=0.7±0.1 nm and mean value=0.6±0.1 nm (RMS=1.2±0.1 and mean value=0.9±0.1 nm).

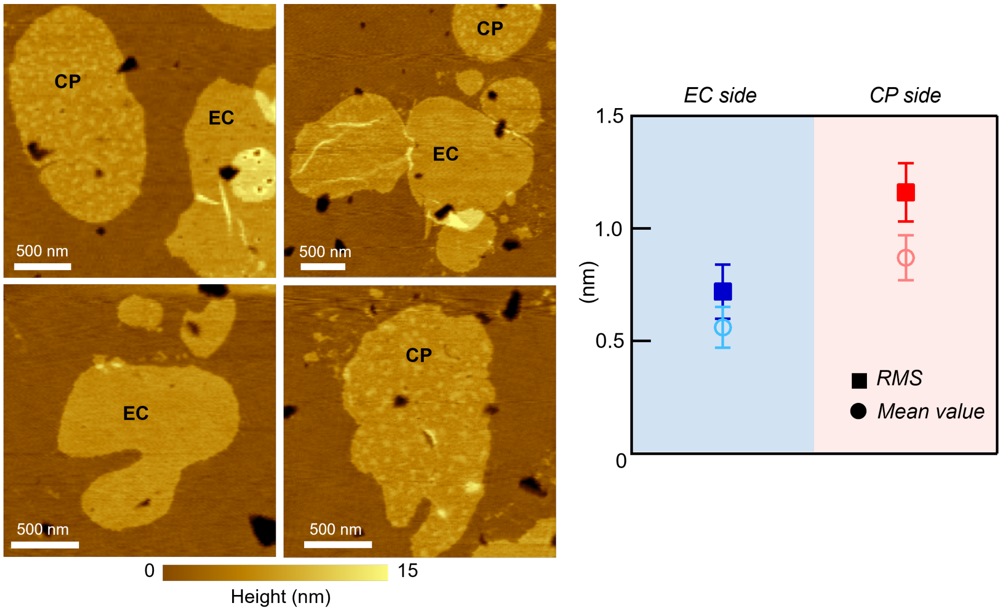

Figure S 5: Left: representative AFM images of individual purple membranes. Right: Average RMS and roughness obtained for the two different membrane orientations.

S7. Comparison between single purple membranes and stacks of purple membranes

In the main text we focused on stacks of purple membranes because we obtained more reliable data compared to single membranes mainly due to the better mechanical stability of the stacks (we experimentally observed that prolonged contact-mode AFM measurements of 5 nm-thick membrane-monolayer patches may lead to a degradation of our sample, hence affecting the AFM-IR signal). However, by comparing the data on stacks with the best data acquired on single purple membranes an overall agreement can be observed. In Figure S6 we compare the difference spectra obtained at +/- 1 V on single purple membranes of opposite orientation (EC-up and CP-up orientation) with the data reported in Figure 3 of

the manuscript, obtained at +/- 3V on a stack of two purple membranes both with CP-up orientation. In Figure S6a we report the difference spectra obtained with the electric field oriented from the EC side to the CP one. In all three cases, we clearly obtain a dip centered at the frequency of the amide-I band maximum. For the opposite electric field direction (S6b), a lineshape more resembling a frequency-shift is obtained. This is more evident for the stack of purple membranes and for the single purple membrane with EC-up orientation. Note that the expected differences for the two orientation directions of BR molecules under a static electric field discussed in the main text (inward and outward the trimer) retain regardless the membrane orientation (EC-up o CP-up). However, the response of BR molecules under a static electric field can be slightly different in the three cases due to other factors such as the presence of the metal surfaces (substrate and cold-coated tip), which, depending on the case, are in contact with different sides of the membrane.

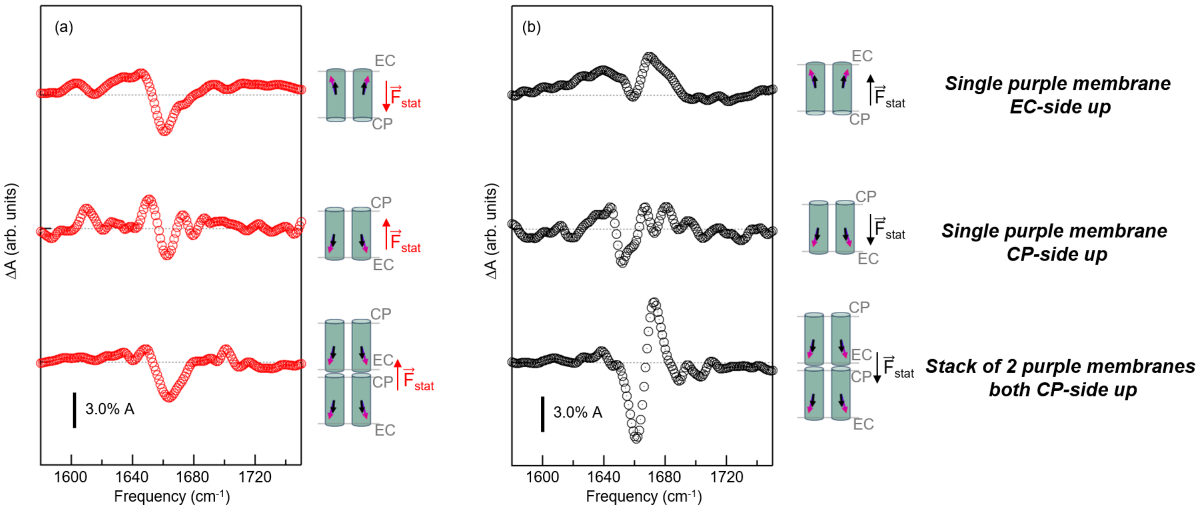

Figure S 6: (a-b) Comparison between the difference spectra obtained at +/- 1 V on single purple membranes of opposite orientation (EC-up and CP-up orientation) with the data reported in Figure 3 of the manuscript, obtained at +/- 3V on a stack of two purple membranes both with CP-up orientation. (a) Electric field oriented form the EC side to the CP one. (b) Electric field oriented form the CP side to the EC one

S8. Current measurements on membranes

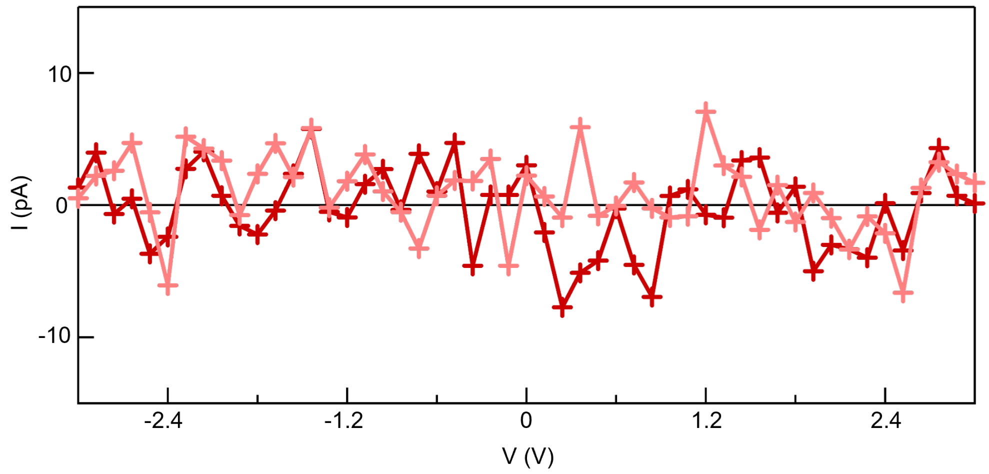

Figure S 7: Two I-V measurements on double membrane patches.

In Figure S7 we report two representative I-V curves measured on double membrane patches in the voltage range between -3V and +3V range to demonstrate that the current is not measurable with our conductive AFM setup. For this measurement we use a platinum tip (by *Rocky Mountain Nanotechonolgy*) suitable for conductive AFM experiments.

S9. Comparison between theoretical VSE and experimental data of Figure 3

According to the theory of VSE, for an anisotropic and immobilized sample, the VSE is expected as a Stark shift, rather than a line broadening.[7] In Figure S8 we plot the expected VSE-shift difference spectra for the two polarities, under the not realistic hypothesis of immobilized sample. To obtain these curves we calculated the difference spectra as follows: $\Delta A(\nu)_{Stark\pm 3V} = A(\nu)_{Stark\pm 3V} - A(\nu)_{0V}$, where $A(\nu)_{0V}$ is the experimental absorption spectrum measured at V=0 V (green curve in Figure3c of the main text), and $A(\nu)_{Stark\pm 3V}$ are equal to the $A(\nu)_{0V}$ with a negative/positive frequency offset. The frequency offset has been calculated according to the relation that defines the Stark shift:[7]

$\Delta\nu = -\vec{F}_{stat} \cdot \Delta\vec{\mu} = \pm F\Delta\mu cos\theta = \pm 3cm^{-1}$, where we used F_{stat}=3 MV/cm, the known Stark tuning rate of C=O bond equal to 1 cm^{-1}/(MV/cm),[8,9] and for simplicity we assumed $\theta = 0°$. It is evident that the VSE theory cannot reproduce the combined $V_i = \pm 3$V experimental data, which, on the contrary, do not show opposite difference spectra for the two polarities. This result is expected given the large freedom of orientation of BR molecules and, hence, the impossibility to interpret the data in the framework of VSE.

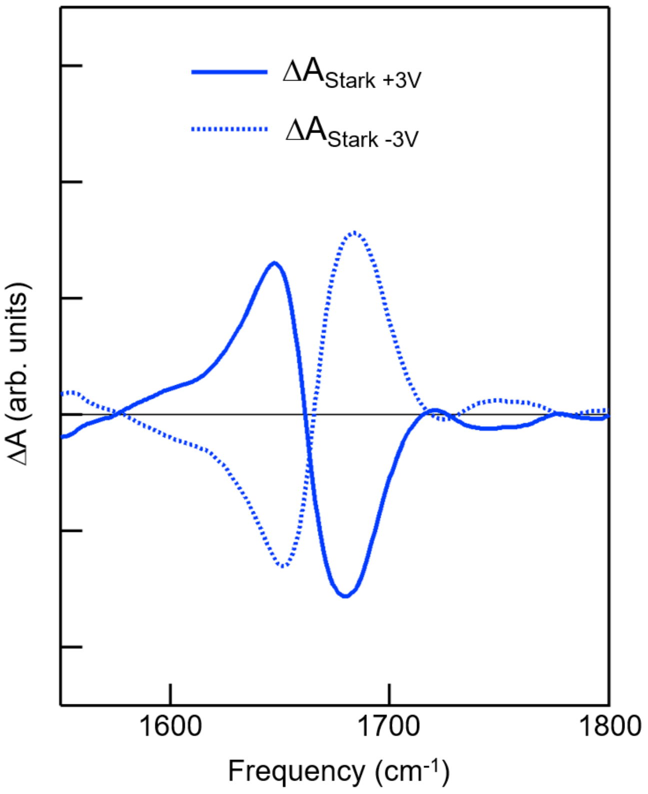

Figure S 8: Expected VSE shift of $\pm 3cmV^{-1}$ of the amide-I band, with opposite sign for the opposite bias.

S10. Phenomenological model

In Figure S9a-c we report sketches of the orientation of the static dipole moment $\vec{\mu}_{stat}$ and of the total C=O dipole moment $\vec{\mu}$ for BR molecules arranged in a trimer. The direction and

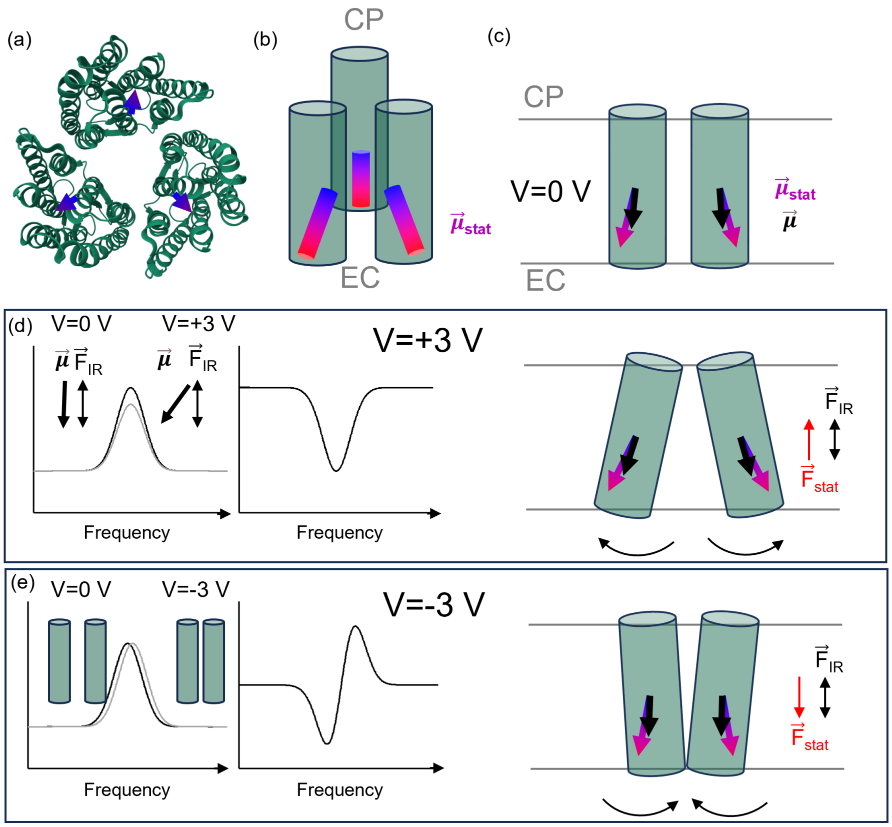

Figure S 9: (a) Structure of BR trimer (image from the RCSB PDB of PDB ID 1FBB,[10] all data and images are available under the CC0 1.0 Universal Public Domain Dedication) where we superimposed the static dipole moment $\vec{\mu}_{stat}$ for each BR sub-unit. (b) Sketch of BR trimer where each green cylinder is a BR sub-unit. (c) Sketch of $\vec{\mu}_{stat}$ and $\vec{\mu}$ for two representative BR proteins belonging to the same trimer. (d-e) Sketch of the proposed phenomenological model for V=+3 V (d) and V=-3 V (e).

versus of $\vec{\mu}_{stat}$ have been obtained using the software described in ref.[11] with PDB-ID Code 1FBB and that of $\vec{\mu}$ from ref.[12] Given the direction of $\vec{\mu}_{stat}$, for positive bias, BR proteins tend to rotate outward from the center of the trimer, with extent of the orientation limited by steric hindrance. The negative dip in the difference spectrum obtained for V=+3 V can then be interpreted as an increase of the relative angle between $\vec{\mu}$ and $\vec{F}_{IR}$ (vertical IR electric field), which, according to the Fermi's golden rule, is estimated to be around 1 degree for $\Delta A(\nu)_{3V} \sim 3\%$ (we assumed an angle in resting condition equal to 39°, according to ref[12]). Due to the small perturbation, one may expect to obtain an opposite difference spectrum for

the opposite bias. However, the effect of BR molecule orientation upon an external electric field is likely to be different for the two polarities mainly due to the offset position of the center of the electric dipole moment towards the EC side with respect to the center of mass of the protein, and to the different steric hindrance in the two directions. Following previous works where inter-molecular interactions in BR has been investigated with computational[13] and experimental approaches,[14–16] the frequency shift observed for V=-3 V can be interpreted as an increase of the coupling between the C=O modes of neighboring alpha-helices due to a decrease of the average distance between adjacent BR molecules and/or BR helices. The observed frequency shift of the amide-I band peak of $\sim$9 cm^{-1} is compatible with a close-packing of the helices within each molecule and of the BR sub-units within the trimers.[13] Note that, according to the simulations of ref,[13] the shift depends non-linearly on the inter-molecular distance, and this could explain why we could not resolve a downshift for the opposite polarity.

References

(1) Ciracì, C.; Urzhumov, Y.; Smith, D. R. Far-field analysis of axially symmetric three-dimensional directional cloaks. *Optics express* **2013**, *21*, 9397–9406.

(2) Olmon, R. L.; Slovick, B.; Johnson, T. W.; Shelton, D.; Oh, S.-H.; Boreman, G. D.; Raschke, M. B. Optical dielectric function of gold. *Physical Review B* **2012**, *86*, 235147.

(3) Bublitz, G. U.; Boxer, S. G. Stark spectroscopy: applications in chemistry, biology, and materials science. *Annual review of physical chemistry* **1997**, *48*, 213–242.

(4) Takashima, K.; Furukawa, Y. Vibrational Stark effect (VSE) on the infrared spectrum of a poly (methyl methacrylate) thin film. *Vibrational Spectroscopy* **2015**, *78*, 54–59.

(5) Knapp, H. F.; Mesquida, P.; Stemmer, A. Imaging the surface potential of active purple membrane. *Surface and Interface Analysis: An International Journal devoted to the*

development and application of techniques for the analysis of surfaces, interfaces and thin films **2002**, *33*, 108–112.

(6) Payam, A. F.; Ramos, J. R.; Garcia, R. Molecular and nanoscale compositional contrast of soft matter in liquid: interplay between elastic and dissipative interactions. *ACS nano* **2012**, *6*, 4663–4670.

(7) Boxer, S. G. Stark realities. *The Journal of Physical Chemistry B* **2009**, *113*, 2972–2983.

(8) Andrews, S. S.; Boxer, S. G. Vibrational stark effects of nitriles I. Methods and experimental results. *The Journal of Physical Chemistry A* **2000**, *104*, 11853–11863.

(9) Pollard, B.; Muller, E. A.; Hinrichs, K.; Raschke, M. B. Vibrational nano-spectroscopic imaging correlating structure with intermolecular coupling and dynamics. *Nature Communications* **2014**, *5*, 3587.

(10) Subramaniam, S.; Henderson, R. Molecular mechanism of vectorial proton translocation by bacteriorhodopsin. *Nature* **2000**, *406*, 653–657.

(11) Felder, C. E.; Prilusky, J.; Silman, I.; Sussman, J. L. A server and database for dipole moments of proteins. *Nucleic acids research* **2007**, *35*, W512–W521.

(12) Marsh, D.; Pali, T. Infrared dichroism from the x-ray structure of bacteriorhodopsin. *Biophysical journal* **2001**, *80*, 305–312.

(13) Eeva-Liisa, K.; Andreas, B. Vibrational Coupling between Helices Influences the Amide I Infrared Absorption of Proteins: Application to Bacteriorhodopsin and Rhodopsin. *J. Phys. Chem. B* **2012**, *116*, 4448–4456.

(14) Torres, J.; Sepulcre, F.; Padros, E. Conformational changes in bacteriorhodopsin associated with protein-protein interactions: a functional. alpha. I-. alpha. II helix switch? *Biochemistry* **1995**, *34*, 16320–16326.

(15) Heyes, C. D.; El-Sayed, M. A. Thermal properties of bacteriorhodopsin. *The Journal of Physical Chemistry B* **2003**, *107*, 12045–12053.

(16) Wang, J.; Heyes, C. D.; El-Sayed, M. A. Refolding of thermally denatured bacteriorhodopsin in purple membrane. *The Journal of Physical Chemistry B* **2002**, *106*, 723–729.
